# Correlation Between Ultrasound-Derived Diaphragmatic Thickness, Inspiratory Muscle Strength and Peak Expiratory Flow in Institutionalized Older Adults: A Cross-Sectional Exploratory Study

**DOI:** 10.3390/geriatrics11050151

**Published:** 2026-10-21

**Authors:** Silvana L. Oliveira-Sousa, Felipe J. Portillo-Castaño, Luis Fernández-Azorín, Felipe León-Morillas, Antonio T. Ríos-Cortés, Adriana C. de Souza Oliveira

**Affiliations:** 1 Physiotherapy and Disability Research Group, Biomedical Research Institute of Murcia Pascual Parrilla-IMIB, 30120 Murcia, Spain; 2 Department of Physical Therapy, University of Murcia, 30120 Murcia, Spain; 3 Health, Disability, Dependency and Well-Being PhD Program, University of Murcia, 30120 Murcia, Spain; 4 Faculty of Nursing, Catholic University of Murcia, 30107 Murcia, Spain; 5 Faculty of Physiotherapy, Occupational Therapy and Podiatry, UCAM Catholic University of Murcia, 30107 Murcia, Spain; 6 Department of Nursing, Pharmacology and Physical Therapy, Faculty of Medicine and Nursing, University of Córdoba, 14071 Córdoba, Spain; 7 Intensive Care Unit, Santa Lucía University General Hospital, 30202 Cartagena, Spain

**Keywords:** diaphragm thickness, maximal inspiratory pressure, peak expiratory flow, homes for older people

## Abstract

**Background:** Diaphragm thickness may affect both inspiratory and expiratory function. The ultrasound assessment of diaphragm thickness could be a valuable tool for respiratory muscle evaluation in older adults, although additional research is needed to clarify its association with respiratory functional parameters. **Objectives:** The primary objective was to explore the relationship between diaphragm thickness (DT), maximal inspiratory pressure (MIP), and peak expiratory flow (PEF) in institutionalized older adults. A secondary objective was to compare MIP and PEF values between participants with normal and thin diaphragm thickness. **Methods**: A cross-sectional exploratory study was conducted among older adults (n = 36) residing in long-term care facilities (LTCFs) in Spain. DT was measured at the end of maximum inspiration (DTi) and at the end of expiration at rest (DTe) in the supine position. The diaphragm thickening difference (DTD) and diaphragm fraction (DTF) were calculated. A thin diaphragm was defined as a diaphragm thickness (DTe) <1.6 mm in men and <1.4 mm in women. The maximum inspiratory pressure (MIP) was measured using a Powerbreath KH1 device, and the peak expiratory flow (PEF) was measured using Datospir Peak-10. Spearman’s rank correlation coefficient and linear regression models were used to explore the association between diaphragm thickness and respiratory muscle function. Statistical analysis was performed using IBM SPSS Statistics 22.0. **Results:** The mean age of the participants was 82.4 ± 7.9 years (24 females–12 males). Significant correlations were observed among most ultrasound-derived measures. DTi was positively associated with MIP (ρ = 0.403, 95% CI 0.065–0.658; q = 0.033) and PEF (ρ = 0.534, 95% CI 0.235–0.741; q = 0.003), DTe with MIP (ρ = 0.445, 95% CI 0.116–0.686; q = 0.020), and DTD with PEF (ρ = 0.475, 95% CI 0.158–0.703; q = 0.012). Compared with participants with normal diaphragm thickness, those with a thin diaphragm exhibited lower MIP (β = −18.43, 95% CI: −32.76 to −4.18; *p* = 0.013). **Conclusions**: Diaphragm thickness was positively associated with inspiratory muscle strength and peak expiratory flow. Participants with a thin diaphragm demonstrated lower respiratory muscle performance than those with normal diaphragm thickness. However, these findings should be interpreted with caution, and additional studies are needed to confirm these associations.

## 1. Introduction

Respiratory muscle strength is an essential marker of ventilatory capacity and a predictor of overall performance [1,2]. The proper functioning of the respiratory muscles (inspiratory and expiratory) is essential to enable patients to cope with infections or other respiratory diseases. A decline in respiratory muscle function can lead to inadequate ventilation and the impaired clearance of airway secretions [3]. The scientific literature reports a decrease in respiratory muscle strength by 0.8 to 2.7 cmH_2_O per year between the ages of 65 and 85 years [4]. This reduction may be even more marked in institutionalized older adults [5,6,7]. Added to this are age-related reductions in the elastic recoil of the lungs and the compliance of the chest wall, which increase the workload on the respiratory muscles [8,9].

The evaluation of respiratory muscle dysfunction is of great clinical importance in many contexts [10,11]. The measurement of maximal respiratory pressures, used as an indicator of respiratory muscle strength, has been widely employed to assess respiratory muscle dysfunction in a variety of clinical and research settings. Although the assessment of maximal respiratory pressures is a non-invasive, valid, and reliable method for evaluating respiratory muscle strength [12], it depends on patient cooperation, and several attempts are required to obtain valid measurements.

In recent decades, in addition to the assessment of maximum respiratory pressures, the use of ultrasound (US) to assess respiratory muscles has shown exponential growth [13,14,15,16,17]. The ability provided by US to observe the structure and/or movement of respiratory muscles in real time has opened an important avenue for clinicians and researchers regarding respiratory assessment and monitoring. Furthermore, it is a harmless, relatively inexpensive (depending on the type of equipment) and quick method [18,19]. Various ultrasound markers have been explored; however, diaphragmatic muscle thickness has received particular attention [13,20], likely because skeletal muscle thickness has been associated with total muscle mass [21,22,23] and due to its potential influence on respiratory muscle strength. Although the diaphragm is primarily an inspiratory muscle, it contributes to several physiological functions beyond inspiration, including important expiratory tasks such as coughing. The generation of adequate expiratory flow depends largely on a preceding deep and sufficiently effective inspiration [24].

Recent evidence supports the wider use of diaphragm ultrasound in geriatric settings [25]. However, caution is warranted when interpreting diaphragm thickness as a surrogate marker of diaphragmatic function. Diaphragm thickness, as assessed by ultrasound, is fundamentally a structural measure and may not directly correspond to respiratory muscle performance. Studies examining the association between structural thickness measurements and functional outcomes are needed to better understand the clinical relevance of diaphragm ultrasound assessments [25]. Although efforts have been made to demonstrate this relationship in other geriatric contexts [26,27,28,29], research involving institutionalized older adults remains scarce.

This population may particularly benefit from assessment methods that do not rely on participant cooperation, voluntary effort, or the ability to follow complex instructions, such as ultrasound imaging. However, for diaphragm ultrasound to have clinical utility in this setting, its measurements should demonstrate meaningful associations with relevant functional outcomes and potentially serve as surrogate markers of respiratory performance. Therefore, studies investigating the relationship between structural diaphragm measurements and functional respiratory parameters are warranted, as they may provide a foundation for establishing the role of diaphragm ultrasound as a marker of respiratory function in older adults.

Therefore, the present study aimed to explore the associations between diaphragm thickness and respiratory muscle function, as assessed by maximal inspiratory pressure (MIP) and peak expiratory flow (PEF), in institutionalized older adults. A secondary objective was to compare MIP and PEF values between participants with normal and reduced diaphragm thickness at rest during end-expiration. We hypothesized that greater diaphragm thickness would be associated with higher respiratory muscle strength and peak expiratory flow. We further hypothesized that participants with reduced diaphragm thickness at end-expiration (selected as the primary exposure variable because it reflects resting diaphragm muscle mass) would exhibit lower MIP and PEF values than those with normal diaphragm thickness.

## 2. Materials and Methods

### 2.1. Study Design

A cross-sectional exploratory study was conducted among institutionalized older adults residing in five public long-term care facilities (LTCFs) in the Region of Murcia, Spain. In accordance with ethical standards, the Research Ethics Committee of the University of Murcia approved the study protocol (approval number: 4170/2023). All participants provided written informed consent after being informed of the study objectives and procedures. The protocol of this study was registered at ClinicalTrials.gov (Identifier: NCT06130800).

### 2.2. Participants

Inclusion criteria were: institutionalized older adults (aged >65 years) who were not bedridden, were able to understand and follow simple verbal instructions (e.g., taking a deep breath and blowing forcefully) to perform the testing maneuvers, and were able to achieve oral occlusion. Exclusion criteria included: chronic respiratory disease, use of inhalers and/or supplemental oxygen, history of respiratory infection within the past six months, severe and/or uncontrolled cardiac disease, presence of a pacemaker, uncontrolled arterial hypertension, neurological disorders or sequelae (e.g., hemiplegia), bronchopulmonary tumors, psychiatric disorders, total hearing or visual loss, and participants who underwent percutaneous endoscopic gastrostomy or had recent postoperative status.

Participant selection was conducted in two phases. First, a nurse from each nursing home performed a preliminary screening by reviewing the medical records of the potential participants and applying this study’s inclusion and exclusion criteria. Subsequently, two research nurses independently reviewed the medical records of the pre-selected individuals to confirm eligibility. They then conducted face-to-face interviews to assess participants’ ability to follow simple commands and performed a visual assessment of oral occlusion.

### 2.3. Main Variables

#### 2.3.1. Diaphragm Ultrasonography

Diaphragmatic thickness (DT) was assessed using B-mode ultrasonography with an Alpinion ECUBEi7™ ultrasound system (Alpinion Medical Systems Co., Ltd., Seoul, Republic of Korea) equipped with a 7–18 MHz linear-array transducer. All ultrasound assessments were performed by the same physiotherapist with experience in musculoskeletal ultrasound. In addition, they received specific training in four 1 h sessions on this study’s measurement protocol.

**Figure 1:**
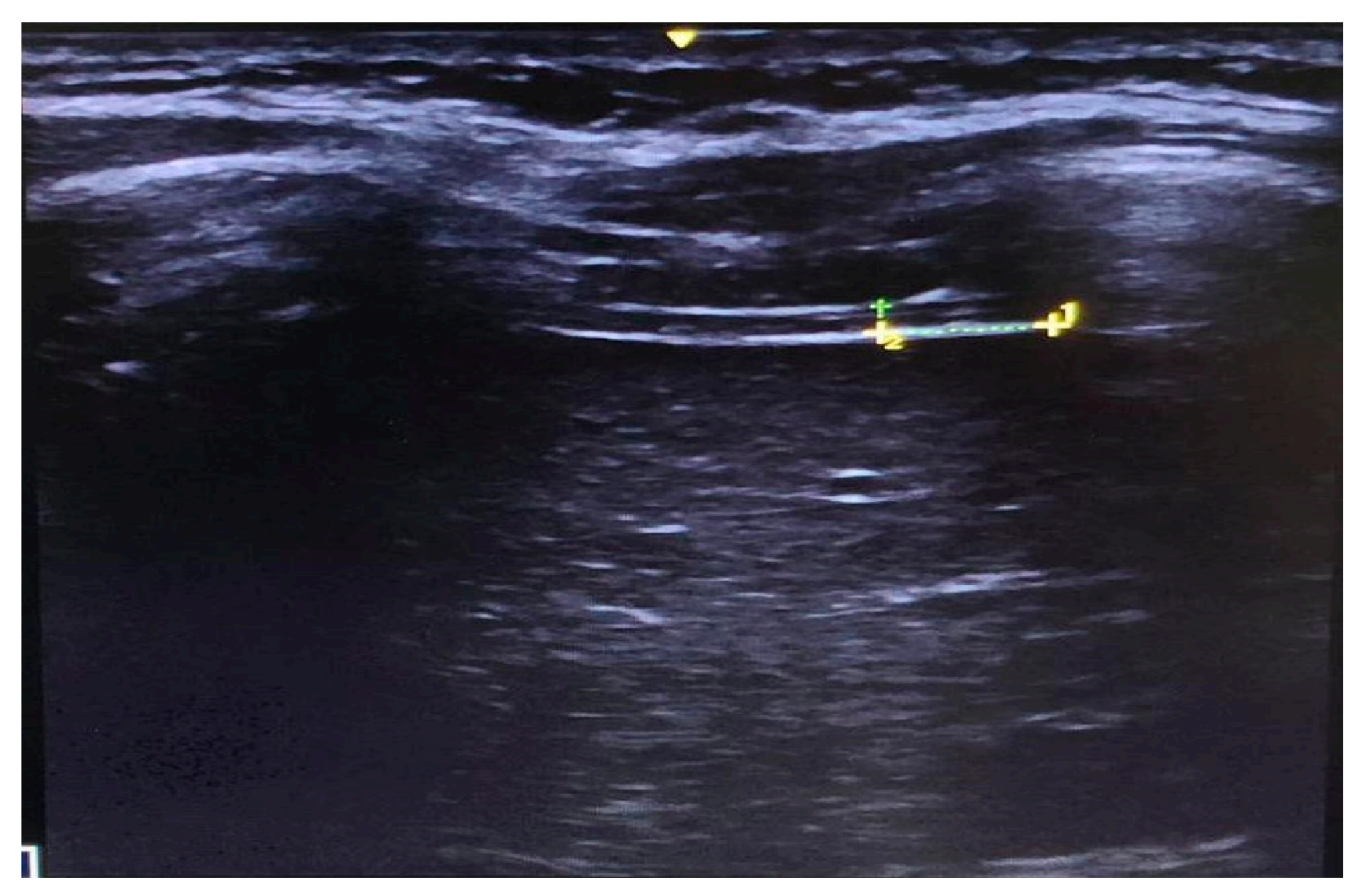
Diaphragm thickness at end-expiration at rest.

The following optimization parameters were applied: 9 MHz frequency, 4.9 cm depth, a dynamic range (DN) of 218, and standard X-contrast mode. Participants were positioned supine, with slight flexion of the lower limbs (using a 14 cm pillow under the popliteal fossa) and head elevation (using an 18 cm triangular cushion) to ensure comfort. As measurements were taken from the right hemithorax, the right arm was abducted to 30–50°. The probe was placed perpendicular to the thoracic wall between the anterior and mid-axillary lines, initially targeting the 7th to 9th intercostal spaces. Participants were instructed to perform a maximal deep inspiration (TLC) to identify the optimal lung window and position the ultrasound probe for image acquisition during both deep inspiration and resting expiration (FRC). The probe was then maintained at the same anatomical location, without being lifted from the participant’s skin, while three images were acquired during deep inspiration and three during resting expiration, in this order. Diaphragm thickness was consistently measured at a standardized location, 0.5 cm from the edge of the last visible rib and outside the pulmonary acoustic window (Figure 1). Thickness was defined as the distance between the inner borders of the pleural and peritoneal membranes and was measured perpendicular to the orientation of the diaphragm muscle fibers. This protocol was previously established in our earlier study [30].

DT was measured at the end of maximal inspiration (DTi) and at the end of expiration at rest (DTe), in that order. Three images were obtained for each phase, and the mean value was calculated. Subsequently, thickness change rates were obtained from these measurements. The diaphragmatic thickening difference (DTD) was calculated by subtracting the end-expiratory thickness at rest (DTe) from the thickness at the end of maximal inspiration (DTi), and the diaphragmatic thickening fraction was calculated using the following formula: DTi − DTe/DTe × 100.

To determine the presence of a “thin diaphragm,” the cut-off values for DTe proposed by van Doorn et al. [31] were applied: <1.6 mm for males and <1.4 mm for females. Normative reference values for diaphragm thickness are not currently available for the Spanish population. Therefore, we selected the sex-specific thresholds proposed by van de Doorn et al. [31], as they were derived from a European cohort including older adults, making them more comparable to our study population than thresholds obtained from younger cohorts or other geographical regions. In addition, the ultrasound acquisition protocol, particularly participant positioning during assessment, was highly consistent with our methodology, supporting the applicability of these cut-off values to our sample.

#### 2.3.2. Maximal Inspiratory Pressure

MIP was assessed using a digital POWERbreathe KH1 device (POWERbreathe International Ltd., Southam, UK). The device incorporates a calibrated electronic pressure sensor and reports MIP as the highest 1 s average inspiratory pressure generated during the maneuver, in accordance with the recommendations of the American Thoracic Society/European Respiratory Society [12]. The pressure measurement range extends from 0 to 240 cmH_2_O, with an accuracy of ±3 cmH_2_O and a precise variation of up to 1 cmH_2_O.

Participants were seated comfortably in a chair with their feet flat on the floor, back supported, and trunk positioned at a 90° angle relative to the hips. They were instructed to exhale maximally (approaching residual volume), followed by a maximal inspiratory effort (approaching total lung capacity) to assess MIP. Strong verbal encouragement was provided during the maneuver, but no visual feedback was given. A nasal clip was used throughout the procedure. Each participant performed a minimum of three efforts, with 1–2 min of rest. Prior to testing, participants performed a familiarization trial to ensure they understood the procedure. The highest value among the three best attempts—with a difference of no more than 5 cmH_2_O—was recorded as the MIP value [32].

#### 2.3.3. Peak Expiratory Flow (PEF)

Peak expiratory flow (PEF) was measured using the Datospir Peak-10 device (Sibelmed, Barcelona, Spain). Participants adopted the same position as previously described. To minimize air leakage and coordination difficulties, participants were instructed to place the mouthpiece in their mouth, take a deep inhalation, and then exhale forcefully and rapidly. This maneuver was performed while wearing a nasal clip [30]. Similarly to the MIP assessment, participants performed at least three attempts, including an initial familiarization trial, with 1 to 2 min of rest between attempts. The highest value achieved was recorded, provided that the difference between the three best measurements did not exceed 20 L/min [33].

To avoid inter-observer errors, all measurements were taken by the same trained investigator. Respiratory assessments were conducted in a standardized order, with MIP measured before PEF.

### 2.4. Secondary Variables

Information regarding comorbidities, sensory impairments, medication use, functional status (Barthel Index; range: 0–100 points), and cognitive status (Mini-Mental State Examination; range: 0–30 points), as well as sociodemographic and anthropometric data, was obtained either from the participants’ medical records or through face-to-face interviews conducted by a nurse with extensive experience in geriatric care.

### 2.5. Statistical Analysis

The IBM SPSS Statistics 22^®^ program was used to record and analyze the data. Quantitative variables were analyzed using the mean, range, median, and interquartile range. Frequencies and percentages were used to describe the qualitative variables. Unadjusted correlation analyses between ultrasound variables and MIP and PEF were performed using Spearman’s rank correlation coefficient (ρ), with corresponding 95% confidence intervals (95% CIs). To account for multiple comparisons, *p*-values were adjusted using the Benjamini–Hochberg false discovery rate (FDR) procedure, considering a total of 15 statistical tests. Results with q < 0.05 are considered significant. Correlation strength was interpreted as follows: negligible, 0.00–0.10; weak, 0.10–0.39; moderate, 0.40–0.69; strong, 0.70–0.89; and very strong, 0.90–1.00 [34].

The Mann–Whitney U test was used to compare PIM and PEF values between participants with a thin diaphragm and those with normal diaphragm thickness. Effect size was quantified using Cliff’s delta. Fisher’s exact test and the Mann–Whitney U test were used to compare categorical and continuous participant characteristics between the two groups, respectively. A univariate linear regression analysis was initially performed to assess the association between thin diaphragm and each outcome variable (MIP and PEF). Subsequently, three separate multivariable models were developed, each including a maximum of three covariates from one domain (sociodemographic, anthropometric, or functional/cognitive), to avoid model overfitting given the small sample size [35]. In Model 1, we included age; in Model 2, weight and height; and finally, in Model 3, the Barthel Index and the MMSE score. Statistical significance was set with a value of *p* < 0.05.

## 3. Results

**Figure 2:**
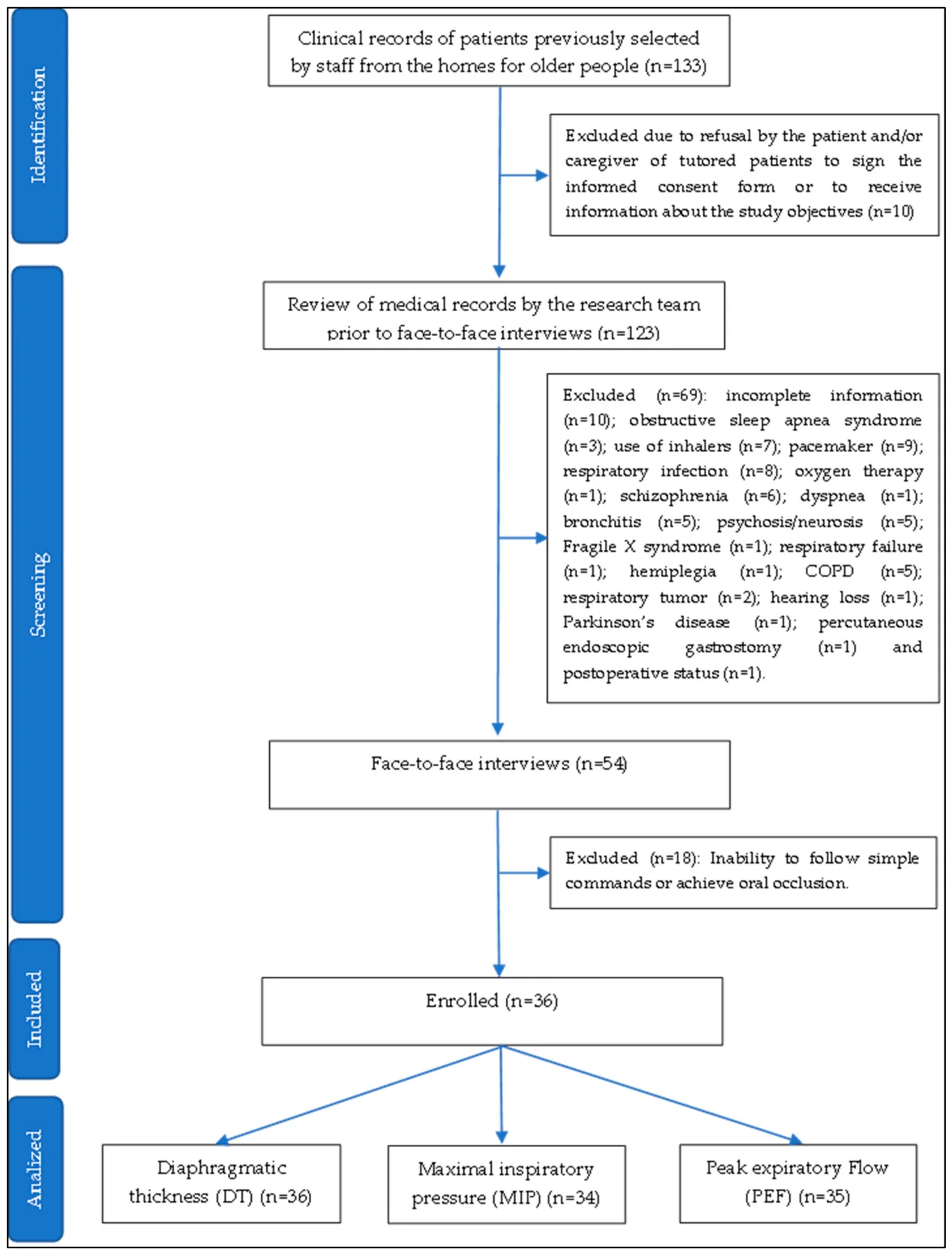
Flow chart of selection of participants.

Staff members from the LTCFs initially reviewed 133 participant medical records for possible inclusion in this study. However, 10 subjects or their caregivers declined participation. Consequently, the research team screened 123 individuals, of whom 36 met the eligibility criteria and were enrolled. The participant identification and selection process is shown in Figure 2.

A total of 36 subjects (66.7% female) were enrolled in this study (Table 1). Participants ranged in age from 67 to 94 years, with a mean age of 82.4 ± 7.9 years. The mean MMSE score was 22.8 ± 5.4, while the mean Barthel Index score was 71.5 ± 28.2.

Descriptive statistics for diaphragmatic thickness and respiratory muscle function are presented in Table 2. The mean diaphragmatic thickness observed at maximum inspiration (DTi) was 3.2 ± 1.0 mm, and that at rest during expiration (DTe) was 1.9 ± 0.6 mm. The maximum inspiratory pressure (MIP) and peak expiratory flow (PEF) reached low mean values of 28.5 ± 18.8 cmH_2_O and 237.8 ± 94 L/min, respectively.

Significant correlations were observed among all ultrasound-derived measures, except between diaphragm thickness at expiration at rest (DTe) and the diaphragm thickness difference (DTD). Both inspiratory and expiratory diaphragm thickness were positively associated with MIP and PEF, although the association between DTe and PEF lost significance after FDR adjustment. Among the ultrasound indices, only DTD showed a significant correlation with PEF. In general, the confidence intervals were wide, indicating considerable uncertainty around the estimated associations (Table 3).

Seventy-five percent (n = 27) of participants had a standard diaphragm, while 25% (n = 9) had a thin diaphragm. Although a higher proportion of women exhibited a thin diaphragm (67% vs. 33%), this difference was not statistically significant (*p* = 1.000). Furthermore, participants with a thin diaphragm did not differ significantly from those with a standard diaphragm in any of the demographic or clinical characteristics assessed.

Participants with a thin diaphragm exhibited significantly lower mean MIP than those with normal diaphragm thickness (14.37 [IC 95% 5.17–23.57] vs. 32.80 [IC 95% 25.21–40.40] cmH_2_O, respectively; *p* = 0.013). The effect size was large (Cliff’s δ = −0.644, 95% CI: −0.933 to −0.274), indicating substantially lower PIM values in the thin diaphragm group.

The association between a thin diaphragm and lower MIP remained significant after adjustment for covariates. In contrast, although participants with a thin diaphragm showed a tendency toward lower PEF values, no significant differences were found between the groups (186.11 [IC 95% 101.36–270.85] vs. 255.76 [222.37–289.16] L/min, respectively; *p* = 0.054). The results of the regression analysis are presented in Table 4.

## 4. Discussion

This study aimed to explore the associations between ultrasound-derived diaphragm thickness, inspiratory muscle strength (MIP), and peak expiratory flow (PEF) and to compare respiratory performance between institutionalized older adults with and without a thin diaphragm. Although our hypotheses could not be confirmed by this study, the findings support the notion that diaphragm thickness may be associated with better respiratory muscle performance. Specifically, ultrasound-derived diaphragm thickness demonstrated positive, moderate associations with inspiratory muscle strength and/or peak expiratory flow. Furthermore, participants with a thin diaphragm exhibited significantly lower inspiratory muscle strength than those with normal diaphragm thickness. However, these findings should be interpreted with caution considering several limitations, which are discussed below.

The ultrasound assessment of the respiratory muscles has become increasingly widespread across a variety of clinical settings and has gained considerable relevance in geriatric populations [25,26,27,28,29]. However, evidence remains scarce among institutionalized older adults. To the best of our knowledge, this is the first study conducted in LTCFs to evaluate diaphragm thickness and its relationship with respiratory muscle function.

As we expected, we observed positive and statistically significant associations between ultrasound-derived diaphragm and the respiratory outcomes examined. Specifically, inspiratory diaphragm thickness was associated with both maximal inspiratory pressure (MIP) and peak expiratory flow (PEF). In contrast, expiratory diaphragm thickness was associated only with MIP, whereas the diaphragm thickness difference (DTD) index was associated exclusively with PEF.

Although diaphragm thickness does not directly reflect diaphragmatic contractile function, evidence from the literature suggests that muscle strength depends on both muscle quality and muscle quantity (e.g., muscle volume, including muscle thickness) [21,22,23]. Therefore, a positive association between diaphragm thickness and strength was expected. Although maximal inspiratory pressure reflects the combined contribution of all inspiratory muscles, including the upper airway muscles, the diaphragm is responsible for the largest proportion of inspiratory force generation during this maneuver. On the other hand, the relationship between diaphragmatic thickness and peak expiratory flow is of particular interest, because PEF is influenced by expiratory muscle recruitment, airway caliber, lung volume, body size, sex, and voluntary effort and is not a direct measure of diaphragm function. However, the PEF maneuver begins with maximal inspiration, and the capacity to achieve high expiratory flows depends partly on the ability of the inspiratory muscles to inflate the lungs adequately before forced expiration. Deep inspiration expands the peripheral airways, improves the mechanical advantage of the expiratory muscles, and increases lung elastic recoil. As a result, PEF reflects the combined contribution of both inspiratory and expiratory muscle strength rather than expiratory muscle performance alone [24].

Nevertheless, several issues should be considered when interpreting these findings. First, the observed associations were moderate rather than strong in magnitude. Second, the wide confidence intervals observed for these estimates indicate a high degree of uncertainty regarding the magnitude of the effects. Therefore, the strength of the associations remains imprecise, and future investigations may yield correlations spanning from weak to strong. Third, the analyses were not adjusted for potential confounding factors, including sociodemographic, anthropometric, cognitive, and functional characteristics. Consequently, the observed associations should be regarded as exploratory and hypothesis-generating. Further studies with larger sample sizes and appropriate adjustment for relevant confounders are needed to confirm these findings and clarify the relationship between diaphragm thickness and respiratory muscle performance in institutionalized older adults.

The scientific literature has not yet reached a consensus regarding the relationship between ultrasound-assessed diaphragm thickness and measures of respiratory function and muscle strength. According to a recent systematic review, stronger associations between diaphragm ultrasound measurements and respiratory functional parameters tend to be observed in populations with marked respiratory muscle impairment, such as patients with neuromuscular disorders or critically ill individuals in intensive care settings [13]. One possible hypothesis is that respiratory muscles possess a substantial physiological reserve, allowing them to maintain adequate function despite structural changes during the early stages of muscle loss. Consequently, reductions in diaphragm thickness may not necessarily translate into measurable declines in respiratory muscle performance in relatively healthy individuals [13]. It is possible that other mechanisms, such as muscle quality and neuromuscular activation mechanisms, may play a more prominent role in force generation [36].

In our study, participants with a thin diaphragm demonstrated respiratory muscle strength values approximately half those of participants with normal diaphragm thickness. Perhaps a considerable reduction in muscle mass may be required before clinically relevant impairments in respiratory muscle strength become evident, highlighting a potential threshold effect in the relationship between diaphragm morphology and function. Although the average diaphragm thickness in our cohort was similar to that reported in community-dwelling older adults [31], the wide range of values observed included exceptionally low measurements, some of which were lower than those documented in previous studies [28,29]. Unfortunately, the limited sample size precluded adjustment for all potential confounding variables through multivariable regression analyses, as the number of participants was at the lower threshold of the recommended participant-to-variable ratio for multivariable modeling; consequently, analyses were limited to subgroup comparisons. On the other hand, we acknowledge that these cut-off values have not yet been validated in institutionalized older adults from Spain. Consequently, the findings should be interpreted with caution, and future studies are needed to establish population-specific normative values and confirm the external validity of these thresholds in Spanish older adults.

Other ultrasound-derived markers have also been investigated for their potential association with respiratory function and muscle strength, yielding promising results [37,38]. In particular, diaphragmatic excursion during maximal voluntary sniffing and excursion velocity during maximal voluntary sniffing have shown stronger associations with respiratory muscle performance than static thickness measurements [37]. Studies conducted in community-dwelling older adults have similarly reported that diaphragmatic excursion, rather than diaphragm thickness, is associated with whole-body sarcopenia [27]. Although the inclusion of additional ultrasound parameters, such as diaphragmatic excursion, could have provided further insights, their assessment was not feasible in the present study. The residents of long-term care facilities follow highly structured daily routines, and the comprehensive respiratory assessment protocol, which already included measurements such as maximal inspiratory pressure (MIP) and peak expiratory flow (PEF), required considerable time and participant engagement. Furthermore, assessment protocols for institutionalized older adults should be as brief and minimally disruptive as possible to maximize feasibility and reduce participant burden.

Although important questions remain unanswered regarding the diagnostic value of diaphragm ultrasound in geriatric settings, it may serve as a useful complement to respiratory muscle assessment. By providing real-time, bedside information on both baseline respiratory muscle status and changes over time, without exposing patients to adverse effects, diaphragm ultrasound could be particularly valuable for monitoring respiratory muscle adaptations following inspiratory muscle training (IMT) interventions [39,40].

### 4.1. Limitations

This study has several limitations that should be acknowledged. First, this study was conducted in a single region of Spain and included a small sample. Although recruitment was undertaken across five residential care facilities, participation was limited, as many residents presented advanced functional and cognitive impairments that precluded their inclusion. Second, diaphragm ultrasound assessments and respiratory function measurements (MIP and PEF) were performed by the same examiner. Although all procedures were conducted following standardized protocols, an intra-rater reliability analysis could not be performed in this study due to time constraints during data collection. Furthermore, MIP and PEF measurements were performed in the seated position, whereas diaphragm ultrasound assessments were conducted with participants in the supine position. Third, the cut-off values used to classify participants as having a thin or normal diaphragm were derived from a Dutch population, as population-specific reference values are not currently available for older Spanish adults. Finally, the cross-sectional observational design precludes any inference of causality between diaphragm thickness, inspiratory muscle strength, and peak expiratory flow.

Therefore, these findings should be interpreted with caution and considered exploratory, providing only preliminary evidence of potential associations. Despite these limitations, we believe that the present results offer valuable insight into the potential role of diaphragm ultrasound assessment in a clinically vulnerable population. If the observed associations between diaphragm thickness, inspiratory muscle strength, and peak expiratory flow are confirmed in larger studies, early screening and targeted intervention strategies could be implemented to identify and manage respiratory dysfunction in this setting.

### 4.2. Future Research

Large prospective studies are needed in institutionalized older adults to confirm the hypotheses generated in this study. Furthermore, studies are needed to establish normative reference values and clarify the prognostic significance of diaphragm ultrasound measures. Future research should also investigate the impact of these ultrasound-derived markers on clinically relevant health outcomes, such as respiratory infections, emergency department visits, hospitalizations, and mortality. In addition, interventional studies will be necessary to determine whether interventions targeting respiratory muscle function can modify diaphragm morphology and improve health outcomes in older adults.

## 5. Conclusions

In this exploratory cross-sectional study of institutionalized older adults, greater ultrasound-derived diaphragm thickness was associated with higher maximal inspiratory pressure and, to a lesser extent, peak expiratory flow. Participants classified as having a thin diaphragm according to previously proposed sex-specific thresholds showed lower respiratory performance, particularly with respect to inspiratory muscle strength. However, these findings should be interpreted cautiously given the small sample size, the limited number of participants in the thin diaphragm subgroup, the cross-sectional design, potential residual confounding, and the lack of population-specific validation of the diaphragm thickness thresholds. Diaphragm ultrasound may represent a potentially useful complementary assessment method, but its clinical screening or diagnostic utility requires confirmation in larger, adequately powered longitudinal studies.

## Figures and Tables

**Table 1:** General characteristics of participants (n = 36).

Variables	Mean ± SD (Range) or n (%)
Sex (female)	24 (66.7)
Age (years)	82.4 ± 7.9 (67–94)
Height (cm)	157.5 ± 9.4 (137.0–180.0)
Weight (kg)	67.0 ± 13.9 (41.3–97.6)
MMSE (0–30)	22.8 ± 5.4 (13–30)
Barthel index (0–100)	71.5 ± 28.2 (25.0–100)
Wheelchair use (yes)	10 (27.8)
Diabetes mellitus (yes)	10 (27.8)
Cardiovascular disease (yes)	14 (38.8)
Artrosis (yes)	15 (41.7)
Osteoporosis (yes)	8 (22.2)
Rheumatoid arthritis (yes)	2 (5.6)
Depression	15 (41.7)
Smoking (yes)	3 (8.3)
Hearing impairment	8 (22.2)
Visual impairment	25 (69.4)
Number of medications	7.6 ± 4.1 (0–17)

MMSE: Mini-Mental State Examination.

**Table 2:** Descriptive statistics of diaphragm thickness and respiratory muscle function.

Variables	Mean ± SD (Range)	Median [IQR]
DTi (mm)	3.2 ± 1.0 (1.1–6.0)	3.0 [2.6–3.7]
DTe (mm)	1.9 ± 0.6 (0.6–3.5)	1.7 [1.5–2.3]
DTD	1.3 ± 0.6 (0.3–3.0)	1.1 [0.7–1.6]
DTF (%)	70.7 ± 35.6 (13.0–148.9)	69.7 [39.4–94.8]
MIP (cmH_2_O)	28.5 ± 18.8 (5–70.0)	25.5 [11.5–42.0]
PEF (L/min)	237.8 ± 94.0 (85–480)	220.0 [165–300]

DTi: diaphragm thickness at maximal inspiration; DTe: diaphragm thickness at expiration at rest; DTD: diaphragm thickness difference; DTF: diaphragmatic thickness fraction; MIP: maximal inspiratory pressure; PEF: peak expiratory flow.

**Table 3:** Correlations between diaphragmatic thickness and respiratory muscle function.

	Rho de Spearman	95% CI	*p*	q (FDR-BH)
**DTi–DTe**	0.665	0.422, 0.818	<0.001	0.003
**DTi–DTD**	0.817	0.662, 0.905	<0.001	0.003
**DTi–DTF**	0.377	0.045, 0.634	0.024	0.040
**DTi–MIP**	0.403	0.065, 0.658	0.018	0.033
**DTi–PEF**	0.534	0.235, 0.741	<0.001	0.003
**DTe–DTD**	0.179	−0.168, 0.487	0.295	0.442
**DTe–DTF**	−0.375	−0.632, −0.043	0.024	0.040
**DTe–MIP**	0.445	0.116, 0.686	0.008	0.020
**DTe–PEF**	0.341	−0.002, 0.612	0.045	0.056
**DTD–DTF**	0.793	0.622, 0.892	<0.001	0.003
**DTD–MIP**	0.351	0.004, 0.622	0.042	0.052
**DTD–PEF**	0.475	0.158, 0.703	0.004	0.012
**DTF–MIP**	0.078	−0.277, 0.414	0.661	0.661
**DTF–PEF**	0.127	−0.225, 0.450	0.467	0.500
**MIP–PEF**	0.430	0.097, 0.676	0.011	0.023

DTi: diaphragm thickness at maximal inspiration; DTe: diaphragm thickness at expiration at rest; DTD: diaphragm thickness difference; DTF: diaphragmatic thickness fraction; MIP: maximal inspiratory pressure; PEF: peak expiratory flow.

**Table 4:** Univariate and multivariate linear regression analysis.

Independent Variables	MIP B Coefficient (IC 95%); Sig.	PEF B Coefficient (IC 95%); Sig.
Thin diaphragm	−18.43 (−32.76 to −4.10); *p* = 0.013	−69.65 (−140.58 to 1.27); *p* = 0.054
Adjusted R^2^	0.177	0.108
**Model 1**		
Thin diaphragm	−17.83 (−31.95 to −3.71); *p =* 0.015	−65.80 (−137.75 to 6.13); *p* = 0.072
Age	−0.56 (−1.35 to 0.21); *p =* 0.150	−1.66 (−5.70 to 2.37); *p* = 0.407
Adjusted R^2^	0.231	0.127
**Model 2**		
Thin diaphragm	−17.03 (−31.42 to 2.63); *p =* 0.022	−47.90 (−109.12 to 13.32); *p* = 0.121
Weight (kg)	0.21 (−0.37 to 0.80); *p =* 0.459	2.04 (−0.516 to 4.60); *p* = 0.114
Height (cm)	26.94 (−51.33 to 105.22); *p =* 0.488	327.68 (−16.67 to 672.04); *p* = 0.061
Adjusted R^2^	0.238	0.400
**Model 3**		
Thin diaphragm	−17.24 (−32.51 to −1.97); *p =* 0.028	−72.82 (−155.69 to 10.04); *p* = 0.083
Barthel index (0–100)	0.260 (0.011 to 0.508); *p =* 0.041	0.423 (−0.997 to 1.84); *p* = 0.546
MMSE (0–30)	0.506 (−0.820 to 1.83); *p =* 0.440	−1.87 (−9.44 to 5.6); *p* = 0.615
Adjusted R^2^	0.370	0.111

MIP: maximal inspiratory pressure; PEF: peak expiratory flow; MMSE: Mini-Mental State Examination.

## Data Availability

The data are not publicly available due to ethical and data protection restrictions. Aggregated information or further details may be made available from the corresponding author upon reasonable request and subject to applicable ethical and data protection requirements.

## Abbreviations

The following abbreviations are used in this manuscript:

DT	Diaphragm thickness
MIP	Maximal inspiratory pressure
PEF	Peak expiratory flow
LTCFs	Long-term care facilities
DTi	DT at the end of maximum inspiration
DTe	DT at the end of expiration at rest
DTD	Diaphragm thickening difference
DTF	Diaphragm fraction
